# PRDM16 regulates arterial development and vascular integrity

**DOI:** 10.3389/fphys.2023.1165379

**Published:** 2023-06-01

**Authors:** Michael Thompson, Masahide Sakabe, Mark Verba, Jiukuan Hao, Stryder M. Meadows, Q. Richard Lu, Mei Xin

**Affiliations:** ^1^ Division of Experimental Hematology and Cancer Biology, Cincinnati Children’s Hospital Medical Center, Cincinnati, OH, United States; ^2^ Department of Pediatrics, College of Medicine, University of Cincinnati, Cincinnati, OH, United States; ^3^ Department of Pharmacological and Pharmaceutical Sciences, College of Pharmacy, University of Houston, Houston, TX, United States; ^4^ Cell and Molecular Biology Department, Tulane University, New Orleans, LA, United States; ^5^ Tulane Brain Institute, Tulane University, New Orleans, LA, United States

**Keywords:** PRDM16, vascular development, angiogenesis, angiopoietin, artery

## Abstract

Proper vascular formation is regulated by multiple signaling pathways. The vascular endothelial growth factor (VEGF) signaling promotes endothelial proliferation. Notch and its downstream targets act to lead endothelial cells toward an arterial fate through regulation of arterial gene expression. However, the mechanisms of how endothelial cells (ECs) in the artery maintain their arterial characteristics remain unclear. Here, we show that PRDM16 (positive regulatory domain-containing protein 16), a zinc finger transcription factor, is expressed in arterial ECs, but not venous ECs in developing embryos and neonatal retinas. Endothelial-specific deletion of *Prdm16* induced ectopic venous marker expression in the arterial ECs and reduced vascular smooth muscle cell (vSMC) recruitment around arteries. Whole-genome transcriptome analysis using isolated brain ECs show that the expression of *Angpt2* (encoding ANGIOPOIETIN2, which inhibits vSMC recruitment) is upregulated in the *Prdm16* knockout ECs. Conversely, forced expression of PRDM16 in venous ECs is sufficient to induce arterial gene expression and repress the ANGPT2 level. Together, these results reveal an arterial cell-autonomous function for PRDM16 in suppressing venous characteristics in arterial ECs.

## Introduction

Vascular formation is regulated by various growth factors, including vascular endothelial growth factor (VEGF), platelet-derived growth factor (PDGF), basic fibroblast growth factor (bFGF), and transforming growth factor-β (TGFβ), which signal to endothelial cells (ECs) through their receptor kinases (Gerhardt et al., 2003; Shih et al., 2003; Ma et al., 2020; Chen et al., 2021). Dysregulation of these signaling pathways may lead to developmental errors during embryogenesis and can cause vascular anomalies, such as capillary malformations or arteriovenous malformations (AVM) (Hellstrom et al., 1999; Cunha et al., 2017; Chen et al., 2021). After the development of the primitive vascular plexus (Goldie et al., 2008), the ECs begin to differentiate into arterial or venous ECs (arterial-venous specification) (Swift and Weinstein, 2009; Kume, 2010). VEGF and Notch signaling play an important role for the regulation of the artery-specific gene expression, whereas COUP-TFII regulates venous-specific gene expression through the inhibition of Notch activity (Krebs et al., 2000; Fischer et al., 2004; You et al., 2005; Gridley, 2007; Swift and Weinstein, 2009). The newly formed arterial ECs then recruit vascular smooth muscle cells (vSMCs) to stabilize the vessels (Gaengel et al., 2009; Stratman et al., 2020). PDGF and angiopoietins are known regulators of vSMC recruitment during vascular development and remodeling (Hellstrom et al., 1999; Gaengel et al., 2009). Mice with genetic loss of *Pdgfb* or its receptor, *Pdgfrb,* die perinatally with vSMC recruitment defects (Levéen et al., 1994; Lindahl et al., 1997). Mice lacking ANGIOPOIETIN1 (encoded by *Angpt1*) or its receptor TIE2 (encoded by *Tek*) show lethality at an early embryonic stage and show a lack of vSMC recruitment, indicating that the ANGIOPOIETIN1-TIE2 signaling pathway plays an important role for vSMC recruitment (Dumont et al., 1994; Sato et al., 1995; Suri et al., 1996). In contrast to the TIE2 activating functions of ANGIOPOIETIN1, ANGIOPOIETIN2 (encoded by *Angpt2*) is known as a functional antagonist of ANGIOPOIETIN1-TIE2 signaling pathway because its overexpression phenocopies *Angpt1* or *Tek* KO mice (Maisonpierre et al., 1997; Gale et al., 2002). However, the molecular mechanisms controlling the expression of these genes remain unclear.

PRDM16 is a member of the PRDM family of transcription factors that are defined by the presence of an active PRDI-BF1-RIZ1 methyltransferase domain (Fog et al., 2012; Pinheiro et al., 2012). PRDM16 has been studied for its role in the regulation of cell fate between muscle and brown fat cells (Seale et al., 2007; Borensztein et al., 2012; Becerril et al., 2013; Cohen et al., 2014; Harms et al., 2014; Li et al., 2015; Gan et al., 2018; Biferali et al., 2021). Also, PRDM16 has roles in craniofacial and cardiac development as well as hematopoietic stem cell maintenance (Bjork et al., 2010; Chuikov et al., 2010; Aguilo et al., 2011; Birjiniuk et al., 2018; Bjork et al., 2018; Corrigan et al., 2018; Cibi et al., 2020; Nam et al., 2020; Shull et al., 2020). Global *Prdm16* knockout mice display respiratory failure and perinatal death (Bjork et al., 2010). In ECs, PRDM16 is highly expressed in the arterial ECs, and is mediated by LMO2 that associates to the *Prdm16* promoter region in zebrafish (Matrone et al., 2021). In mice, PRDM16 is indispensable for the recovery of arterial flow upon femoral artery ligation due to the role of PRDM16 for maintaining endothelial function (Craps et al., 2021). A recent study has shown that PRDM16 is expressed in the retinal arteries of adult mice (Craps et al., 2021); however, a detailed analysis of PRDM16 during development and angiogenesis remains unclear.

Here, we investigate the expression of PRDM16 in developing mouse embryos and retinas. We found that loss of *Prdm16* in ECs induces ectopic venous marker expression and proliferation in the arterial ECs and less vSMC recruitment around the arterial vessels. We also found that the expression of *Angpt2* is upregulated in EC-specific *Prdm16* knockout mice, suggesting that PRDM16 may have a crucial role for proper arterial vascular development.

## Materials and methods

### Animals

All animal experiments were performed with the approval of the institutional animal care and use committee of Cincinnati Children’s Hospital Medical Center. Mouse lines harboring the *Prdm16* floxed alleles, *PDGFb-ER* ^*T2*^ *-Cre* (*PDGFbCre*), and *Tek/Tie2-Cre* (*Tie2Cre*) have been previously described (Kisanuki et al., 2001; Claxton et al., 2008; Cohen et al., 2014). Embryos were collected from pregnant *Prdm16* ^*flox/flox*^ females at the predicted embryonic time point after breeding with a *Prdm16* ^*flox/+*^ *; Tie2-Cre* male mouse. These alleles were kept on mixed backgrounds. Tamoxifen (Sigma) was dissolved in 90% sunflower oil (Sigma) and 10% ethanol to 2 mg/mL. Cre induction by tamoxifen was performed via intraperitoneal (IP) injection from postnatal (P) 1-3, with 0.1 mg tamoxifen injected on each day.

### Whole-mount immunostaining of retinas

Retinas were collected as previously described (Crist et al., 2017). Briefly, neonates were sacrificed by isoflurane overexposure and eyes were removed and fixed with 4% paraformaldehyde (PFA, Thermo Scientific) in phosphate buffered saline (PBS, Fisher Scientific) for 1h at 4°C, then washed with PBS. Retinas were dissected and washed with PBS, permeabilized with 1% Triton-X100 (Fisher Scientific) in PBS for 30 min at RT, then blocked with CAS-Block (Life Technologies) for 30 min at RT. Retinas were incubated with primary antibodies (Supplementary Table S1) or IB4 conjugated with Alexa Fluor 488 (ThermoFisher, 1:100) overnight in 1% Triton-X100 in PBS at 4°C on a rocker. Retinas were washed with PBS, then incubated with secondary antibodies (Supplementary Table S1) for 4 hs at RT on a nutator mixer. Retinas were washed with PBS, then leaflets were cut into the retinas for flat mounting on slides under a coverslip with Fluoromount-G (SouthernBiotech). Images were taken using Eclipse Ti Confocal microscopy with a C2 laser-scanning head (Nikon). Images showing the superficial, intermediate, and deep layers were stacked using ImageJ (Schneider et al., 2012). Depth coded images were prepared using the “Temporal-Color Coder”, provided by ImageJ. ENDOMUCIN and COUP-TFII mean fluorescence images were quantified using ImageJ.

### EdU incorporation

5 μg/kg 5-ethynyl-2′-deoxyuridin (EdU, Invitrogen) was injected into mice at P7 via IP injection. 24 hs later, retinas were collected, and then fixed with 4% PFA, blocked with CAS-Block, and incubated with anti-ERG antibody. EdU was then labeled using the Click-iT EdU 488 imaging kit (Invitrogen). Retinas were then washed in PBS at RT on a shaker, then proceeded to secondary antibody treatment, as previously detailed. To quantify the proliferating ECs, we counted EdU and ERG double positive cells on the main vessel trunk between the optic nerve and 50% vascular extension.

### Isolation of brain ECs

P5 mice were knocked down using ice and decapitated. Brains were removed and placed in ice cold PBS. Enzymes from the Neural Tissue Dissociation Kit (P) (MACS Miltenyi Biotec) were added to the brains and were then digested using the gentleMACS Octo Dissociator with Heaters following the manufacturer’s protocol. The dissociated cell suspension was then incubated with beads conjugated with anti-CD31 antibody (MACS Miltenyi Biotec) and applied to a magnetic column (MACS Miltenyi Biotec). CD31^+^ cells were eluted from the column for RNA isolation.

### RNA isolation and RT-qPCR

RNA from CD31^+^ cells were isolated according to the NucleoSpin RNA XS kit (Macherey-Nagel), then stored at −80°C until analysis. RNA from P9 retinas were isolated using TRIzol (Invitrogen) following the manufacture’s protocol. RNA concentration was assessed by NanoDrop (ThermoFisher). cDNA was generated using PrimeScript RT MasterMix (Takara). Briefly, 50 ng or 500 ng of total RNA for CD31^+^ cells or retinas, respectively, were used according to the manufacturer’s protocol. qPCR was performed with PowerUp SYBR Green Master Mix (applied biosystems) with primers as detailed in Supplementary Table S2. Data was collected on a Step One Plus RT-PCR system (applied biosystems). *Pecam1* served as the internal control for the qPCR analysis of retina and isolated brain EC, while *18s* was used for the internal control for the qPCR analysis of HUVECs.

### RNA sequencing and data processing

PolyA RNA-seq was performed as previously described (Walsh et al., 2019; Rapp et al., 2020). Briefly, total RNA quality was checked by Bioanalyzer (Agilent). RNA was prepared from 100 ng total RNA with NEBNext Poly(A) mRNA Magnetic Isolation Module (NEB), then was enriched with SMARTer Apollo NGS library system (Takara), and the library was prepared using NEBNext Ultra II Directional RNA library prep kit (NEB) with 13 PCR cycles. Libraries were checked for quality and quantified with Qubit (ThermoFisher), then pooled and sequenced with NextSeq 550 sequencer (Illumina) to generate 25 M reads per sample. Fastq files were generated with Illumina BaseSpace Sequence Hub. Differentially expressed genes were identified via BaseSpace RNA-Seq Alignment v2.02, followed by RNA-Seq Differential Expression app v 1.0.1. Reads were aligned to Mus. Musculus/MM10 under 1st strand setting, analyzed using STAR for alignment and Salmon for quantification. Alignment results were analyzed in RNA-Seq Differential Expression app. RNA sequencing was performed by Genomics, Epigenomics and Sequencing Core, University of Cincinnati.

### Cell culture

Human Umbilical Vein Endothelial Cells (HUVECs) were cultured at 37°C, 5% CO_2_ in EGM-2 medium (Lonza).

### Lentivirus generation and infection

*Prdm16* was subcloned from pcDNA3.1-*Prdm16,* a gift from Bruce Spiegelman (Addgene plasmid # 15503 (Seale et al., 2007)), into pLVX lentiviral vector. Lentivirus for overexpression of *Prdm16* was generated as previously described (Sakabe et al., 2017). Briefly, pLVX with or without *Prdm16* was co-transfected into HEK293-FT cells, along with psPAX2 (packaging vector) and pMD2. G (envelope vector), while cells were cultured in DMEM (Invitrogen) with 10% FBS (R&D). Viral supernatants were collected 24 and 48 h post-transfection, pooled together and subjected to ultracentrifugation at 25,000 RPM for 2 h at 4°C. Supernatant was carefully removed and the viral pellet was suspended in 100 µL residual DMEM with 10% FBS overnight at 4°C. HUVECs were infected in a 6-well plate (Falcon) with 10 µL of control or *Prdm16* virus in EGM-2 supplemented to 8 μg/mL Polybrene for 24 h. Infected HUVECs were maintained with EGM-2 and lysed with 2x sample buffer (Bio-Rad) with *β*-mercaptoethanol 4 days post-infection.

### Western blotting

Infected HUVECs were lysed with 200 µL 2x sample buffer with *β*-mercaptoethanol and boiled at 95°C for 5 min. 10µL of protein sample was loaded to a 6% or 10% SDS-PAGE gel, followed by transfer to Immobilon-P PVDF membrane (Millipore). Membranes were briefly washed and then blocked with 5% skim milk in wash buffer for 30 min. Membranes were incubated overnight at 4°C with anti-PRDM16 (R&D, 1:1000), anti-GAPDH (Millipore, 1:3000), or anti-ANGPT2 (R&D, 1:500) antibodies. After washing, membranes were incubated with anti-sheep-HRP (Sigma, 1:1000), anti-mouse-HRP (GE Healthcare, 1:1000), or anti-Goat-HRP (Millipore, 1:5000) secondary antibodies at room temperature for 1.5 h. Membranes were washed and then imaged on a ChemiDoc (Bio-Rad) using SuperSignal West Pico PLUS (ThermoFisher) or combined Pico PLUS and Femto Chemiluminescent Substrates (ThermoFisher, 9:1).

### Statistical analysis

IB4 stained retinas were quantified in Fiji (ImageJ (Schindelin et al., 2012)). Vascular extension was measured by dividing the angiogenic length by the total retina petal length. Branch points and vascular areas were quantified on a 500 µm × 500 µm image in Angiotool (Zudaire et al., 2011). Used animal numbers or group numbers are described in their respective figure legends. We calculated *p*-values with unpaired Student’s t-test with Prism9 (GraphPad). A *p*-value <0.05 was considered to represent a statistically significant difference. Data are presented as mean ± SD.

## Results

### PRDM16 is expressed in the arterial ECs in developing embryonic vessels

**FIGURE 1:**
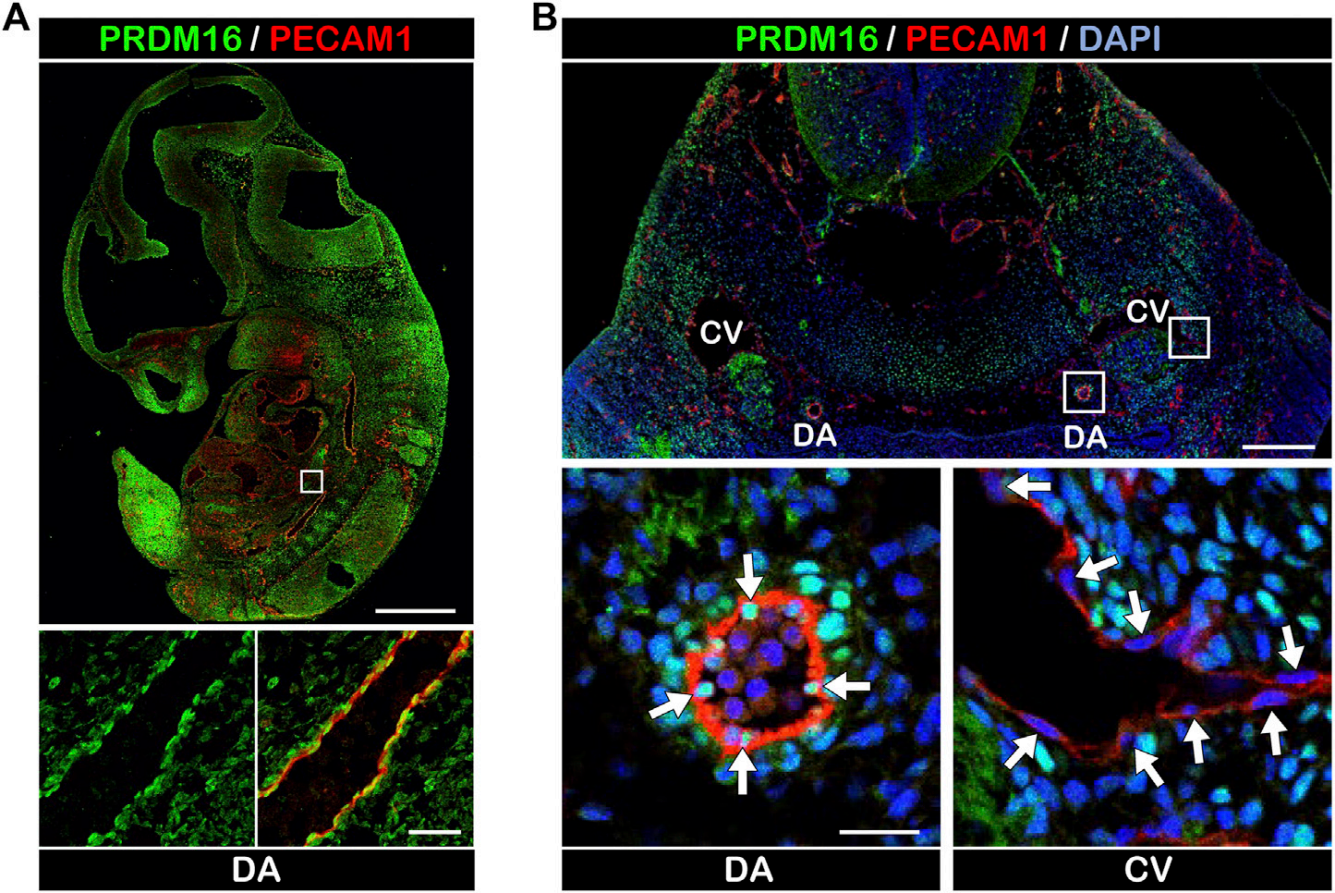
PRDM16 is expressed in arterial ECs in E11.5 embryos **(A)** PRDM16 and PECAM1 immunostaining of sagittal section of mouse embryo at E11.5. PRDM16 (green) can be detected in ECs (PECAM1, red) of the dorsal aorta (lower panel). DA, dorsal aorta. Scale bars, 1 mm (upper panel), 50 µm (lower panel). **(B)** PRDM16 and PECAM1 staining of transverse section of mouse embryo. Higher magnification of DA (left) and CV (right) are shown in the lower panels. DA, dorsal aorta, CV, cardinal vein. Scale bars, 200 µm (upper panel), 20 µm (lower panel).

To investigate the expression of PRDM16 in developing vessels, we performed immunostaining using embryonic day (E) 11.5 wild-type mouse embryos. PRDM16 was detected in several tissues including the heart and somites. Interestingly, PRDM16 was also detected in the PECAM1-positive vascular endothelial cells (ECs) in the dorsal aorta (DA) (Figure 1A). As shown in the transverse section through the pharyngeal arch region, PRDM16 can be detected in the ECs in the DA (arrows in Figure 1B, lower left panel). In contrast, ECs in the cardinal vein (CV) did not show apparent staining of PRDM16 (arrows in Figure 1B, lower right panel). The PRDM16 expression was also observed in the smooth muscle alpha actin (αSMA)-positive vascular smooth muscle cells (vSMCs) (arrow heads in Supplemental Figure S1, lower left panel). These observations suggest that PRDM16 is expressed in the arterial ECs and vSMCs but not in the venous ECs in developing vessels.

### Arterial ECs in the EC-specific *Prdm16* knockout embryos display reduction of arterial characteristics

**FIGURE 2:**
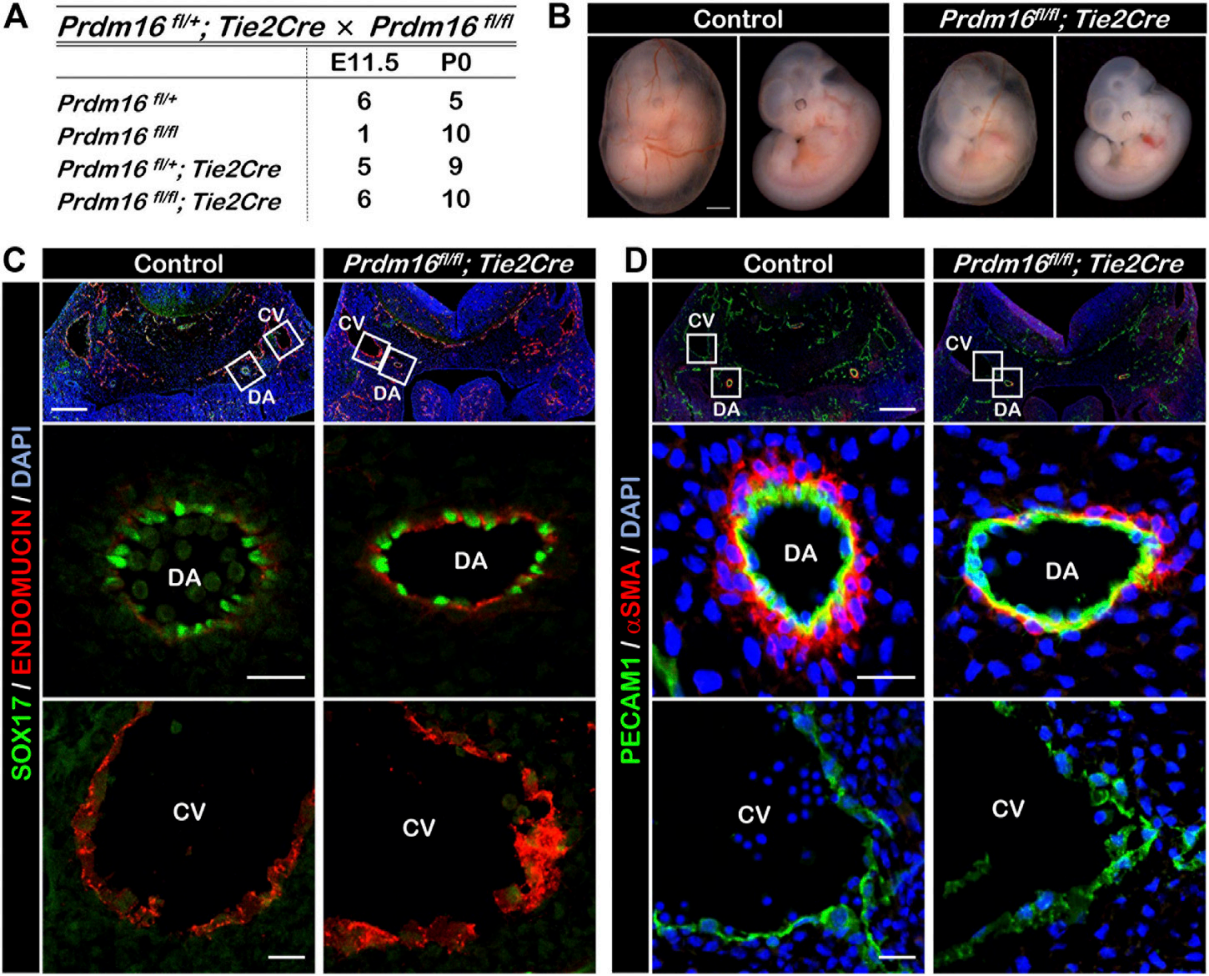
Embryonic deletion of *Prdm16* in ECs causes ectopic ENDOMUCIN expression and reduced vSMC recruitment to arteries **(A)** Summary of genotypes of litters obtained by breeding between *Prdm16* ^*fl/fl*^ female and *Prdm16* ^*fl/+*^ *; Tie2Cre* male mice. Genotyping was performed at E11.5 and P0. No dead embryos or mice were noticed. **(B)** Whole-mount images of E11.5 control and *Prdm16* ^*fl/fl*^ *; Tie2Cre* embryos, with and without yolk sac (left and right panels, respectively). Scale bar, 1 mm. **(C)** Immunostaining of transverse sections of E11.5 control and *Prdm16* ^*fl/fl*^ *; Tie2Cre* embryos for SOX17 (green) and ENDOMUCIN (red). Scale bars, 200 µm (upper panels), 20 µm (middle and lower panels). DA, dorsal aorta, CV, cardinal vein. **(D)** Immunostaining of transverse sections of E11.5 control and *Prdm16* ^*fl/fl*^ *; Tie2Cre* embryos for PECAM1 (green), and αSMA (red). Scale bars, 200 µm (upper panels), 20 µm (middle and lower panels).

To investigate the role of PRDM16 in the ECs during development, we bred *Prdm16* ^*fl/fl*^ mice with the *Tie2Cre* mouse line to generate EC-specific *Prdm16* knockout embryos (*Prdm16* ^*fl/fl*^ *; Tie2Cre*). The loss of *Prdm16* in ECs did not cause any changes to the expected Mendelian ratios at postnatal day (P) 0 (Figure 2A). The vascular formation in the yolk sac of *Prdm16* ^*fl/fl*^ *; Tie2Cre* embryos appeared to be normal at E11.5, but the knockout embryos seemed to be smaller than the control embryos (Figure 2B). We investigated whether deletion of *Prdm16* can affect arterial characteristics as we have shown that PRDM16 is specifically expressed in arterial ECs. Immunostaining analysis showed that the expression of the arterial marker SOX17 was comparable in ECs of DA between of the control and *Prdm16* mutant embryos (Figure 2C). The venous EC marker, ENDOMUCIN, was expressed only in the CV but not in the DA of control embryos, whereas we observed ectopic expression of ENDOMUCIN in the DA of *Prdm16* ^*fl/fl*^ *; Tie2Cre* embryos (Figure 2C). We next investigated the vSMC recruitment because the arterial ECs start to recruit the vSMCs around E10.5, which is one of the characteristics of arterial ECs. The DAs of control embryos were surrounded by a thick layer of αSMA-positive vSMCs, whereas only a few vSMCs were observed in the DAs of the *Prdm16* ^*fl/fl*^ *; Tie2Cre* embryos (Figure 2D). These results suggest that loss of *Prdm16* in developing ECs leads to a decrease of arterial characteristics with increased venous marker expression in the arterial ECs of *Prdm16* ^*fl/fl*^ *; Tie2Cre* embryos.

### PRDM16 is expressed in arterial ECs of the retina

**FIGURE 3:**
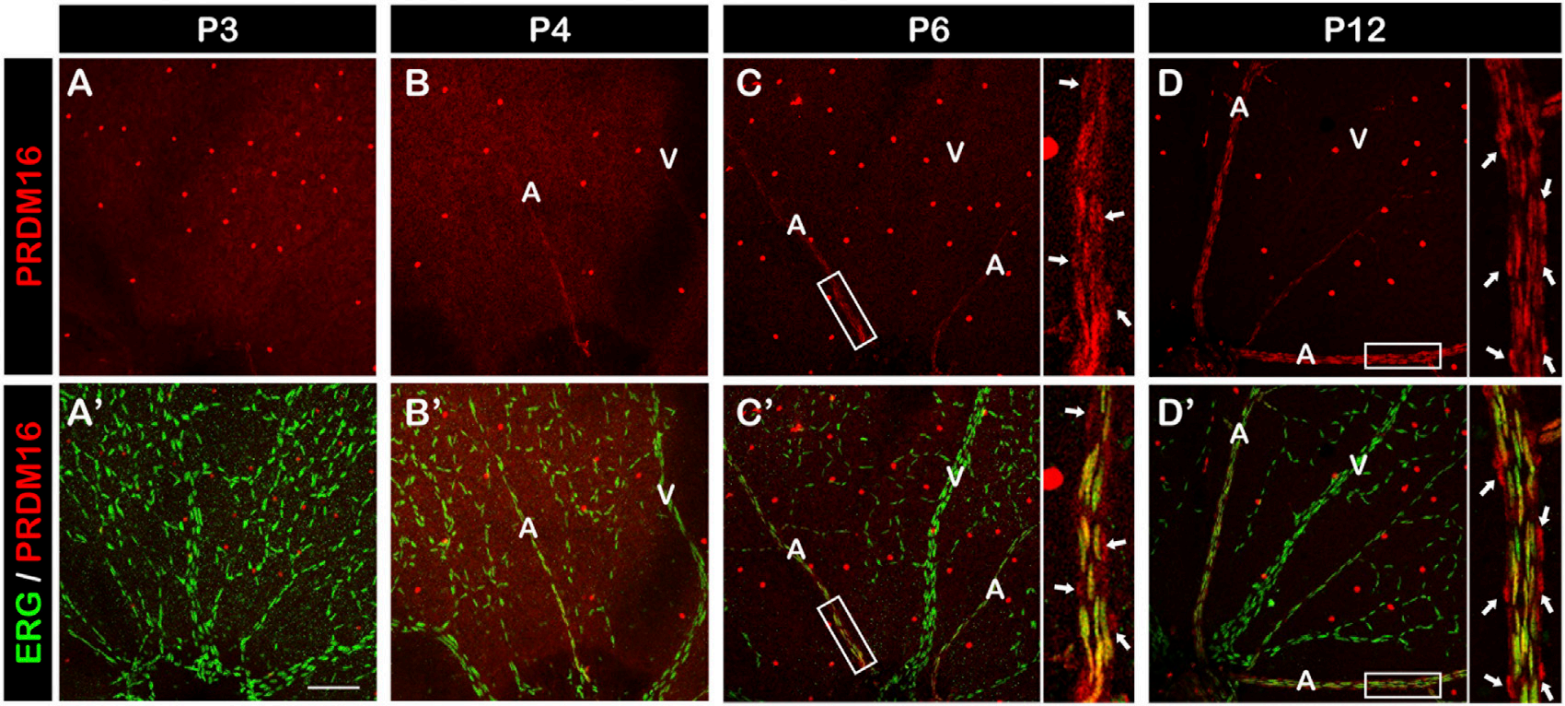
PRDM16 expression in developing mouse retina. Immunostaining of PRDM16 (red) and ERG (green) in whole-mount retina at the indicated time points for P3 **(A, A′)**, P4 **(B, B′)**, P6 **(C, C′)**, and P12 **(D, D′)**. Insets in **(C, C′)**, **(D, D′)** show high-magnification images of arteries in the P6 and P12 retina. Arrows in D and D′ indicate likely vSMCs surrounding artery that are known to express PRDM16. A: Artery. V: Vein. n = 3. Scale bar, 100 µm. Note that bright red dots throughout retinas are ganglion cells.

We next investigated the role of PRDM16 in postnatal angiogenesis using the mouse retina as a model system. The retinal vessels start to develop at birth, allowing for a time course analysis of vascular formation. At P3, premature retinal vessels were already formed, but we didn’t observe PRDM16 expression in ECs (Figure 3A, A′). By P4, arteriovenous specification has occurred and, interestingly, PRDM16 started to be expressed in the arterial ECs but not in the venous ECs (Figure 3B, B′). From P6, when arterial ECs start to recruit vSMCs, PRDM16 can be detected in both arterial ECs and vSMCs (Figure 3C, C′) and the same expression pattern persisted at a later time point during retinal angiogenesis (Figure 3D, D′) (Jeong et al., 2017). Note that PRDM16 was also expressed in the ganglion cells (bright dots in each retina) (Su et al., 2020). This time course expression analysis indicates that PRDM16 starts to be expressed in the retinal arterial ECs at the beginning of arteriovenous specification.

### Loss of *Prdm16* prevents endothelial migration in developing retinal vessels

**FIGURE 4:**
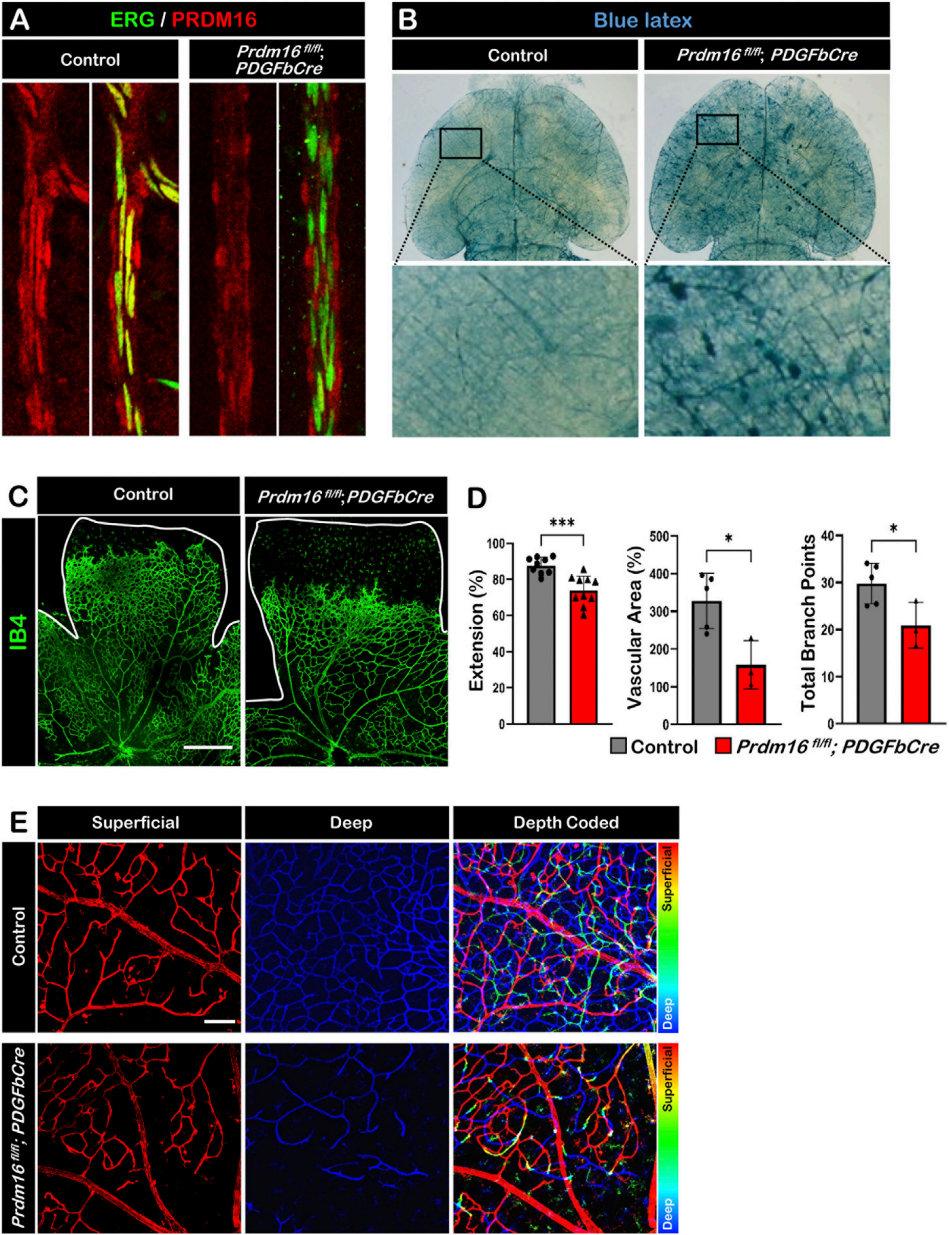
Deletion of *Prdm16* results in retina vascular defects **(A)** PRDM16 (red) and ERG (green) co-immunostaining of control and *Prdm16* ^*fl/fl*^ *; PDGFbCre* mouse retinas at P12 to show *Prdm16* deletion efficiency. **(B)** Gross image of P9 brains injected with blue latex to visualize vasculature. Insets show normal vasculature in the control mice (left), while vascular abnormalities are seen in the *Prdm16* ^*fl/fl*^ *; PDGFbCre* mice (right). **(C)** Whole mount IB4 staining of P8 control and *Prdm16* ^*fl/fl*^ *; PDGFbCre* retina. n > 3. Scale bar, 500 µm. **(D)** Quantification of the vasculature extension (control n = 9, *Prdm16* ^*fl/fl*^ *; PDGFbCre* n = 10), vascular area (control n = 5, *Prdm16* ^*fl/fl*^ *; PDGFbCre* n = 3), and total branch points (control n = 5, *Prdm16* ^*fl/fl*^ *; PDGFbCre* n = 3). Data are as mean ± SD, *: *p* < 0.05, ***: *p* < 0.001 (Student’s t-test). **(E)** Representative pseudo-colored images of P12 control and *Prdm16* ^*fl/fl*^; *PDGFbCre* mouse retinas. Vasculature marked with IB4. Superficial and deep plexus images were overlaid using ImageJ, superficial layer in red, changing to blue moving to the deep plexus. Scale bar, 100 µm, n = 3.

To investigate the role of PRDM16 in the developing vasculature, we generated inducible EC-specific *Prdm16* knockout mice (*Prdm16* ^*fl/fl*^ *; PDGFbCre*). Tamoxifen was administered from P1 to P3, and tissues were collected for analysis between P8 and P12. Deletion of PRDM16 in ECs was confirmed by immunostaining, and PRDM16 remained unchanged in vSMCs in *Prdm16* ^*fl/fl*^ *; PDGFbCre* mice at P12 (Figure 4A). To examine the global effect of *Prdm16* loss on the vasculature, we injected blue latex into control and *Prdm16* ^*fl/fl*^ *; PDGFbCre* mice at P9. We found abnormal vascular morphology in the brain of *Prdm16* ^*fl/fl*^ *; PDGFbCre* mice compared to the control mice (Figure 4B). To further investigate the role of *Prdm16* in angiogenesis and any resulting phenotype, we collected retinas from control and *Prdm16* ^*fl/fl*^ *; PDGFbCre* mice at P8 and stained them with IB4 to visualize the vascular plexus. Although PRDM16 is normally expressed only in the arterial ECs, we found significantly less vascular extension, vascular area, and fewer branch points in the superficial layer of *Prdm16* ^*fl/fl*^ *; PDGFbCre* retinas (Figures 4C, D). In the P12 control retinas, some ECs on the superficial layer migrated into the inner retina and generated the deep retinal vascular plexus (Figure 4E). In *Prdm16* ^*fl/fl*^ *; PDGFbCre* retinas, however, we observed a dramatic decrease of deep retinal vascular plexus formation (Figure 4E). Together, loss of *Prdm16* in ECs causes a reduction of vascular migration and disturbed deep plexus formation.

### Loss of *Prdm16* in ECs leads to ectopic venous marker expression in the retinal arteries

**FIGURE 5:**
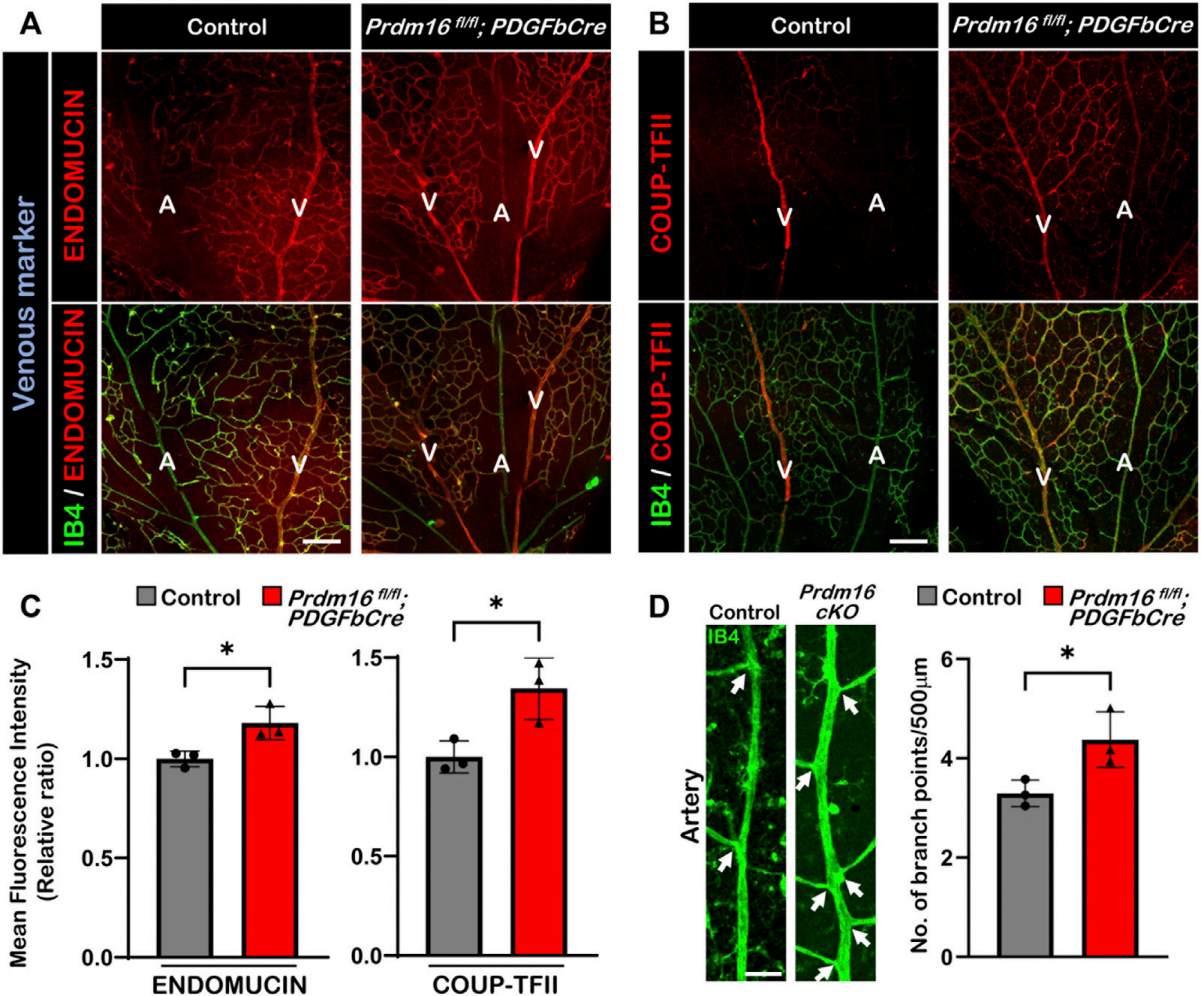
Ectopic expression of venous markers ENDOMUCIN and COUP-TFII in *Prdm16* ^*fl/fl*^; *PDGFbCre* arteries. Immunostaining of whole mount P9 retina with IB4 (green) and venous markers in red (ENDOMUCIN **(A)** or COUP-TFII **(B)**). Scale bar, 100µm, n = 3. **(C)** Quantification of mean fluorescence intensity of ENDOMUCIN and COUP-TFII in *Prdm16* ^*fl/fl*^; *PDGFbCre* mice normalized to the control retinas. n = 3; Data are as mean ± SD, Student’s t-test: *p < 0.05. **(D)** Representative images of P9 retinal arteries in control and *Prdm16* ^*fl/fl*^; *PDGFbCre* (*Prdm16 cKO*) mice (left panel) and quantification of the number of branch points (right panel). Arrows indicate branch points. n = 3; Data are as mean ± SD, Student’s t-test: *p < 0.05.

As we observed ectopic ENDOMUCIN expression in the DAs of *Prdm16* ^*fl/fl*^ *; Tie2Cre* embryos, we next investigated whether venous marker expression was also increased in arterial ECs in *Prdm16* ^*fl/fl*^ *; PDGFbCre* retinas. We found ectopic ENDOMUCIN and COUP-TFII expression in the arterial ECs of *Prdm16* ^*fl/fl*^ *; PDGFbCre* retinas (Figures 5A–C). In line with the ectopic venous marker expression, the *Prdm16* ^*fl/fl*^ *; PDGFbCre* mice showed more branches in the arteries than in control mice, indicating a slight shift towards being more vein like (Figure 5D). To examine if loss of *Prdm16* altered the expression of artery markers, we performed immunostaining for SOX17 and RT-qPCR using RNA from P9 whole retina. No changes were found in SOX17 or the artery markers such as *Cxcr4*, *Dll4*, or *Hrt2* (Supplementary Figures S2A, S2B). Since the phenotypes of the arterial ECs in *Prdm16* ^*fl/fl*^ *; PDGFbCre* retinas are consistent with *Prdm16* ^*fl/fl*^ *; Tie2Cre* embryos, these results strongly suggest that PRDM16 is necessary for proper vascular development and may be involved in regulating arterial specification during the vascular formation.

### Loss of *Prdm16* causes more proliferation in retinal arterial ECs

**FIGURE 6:**
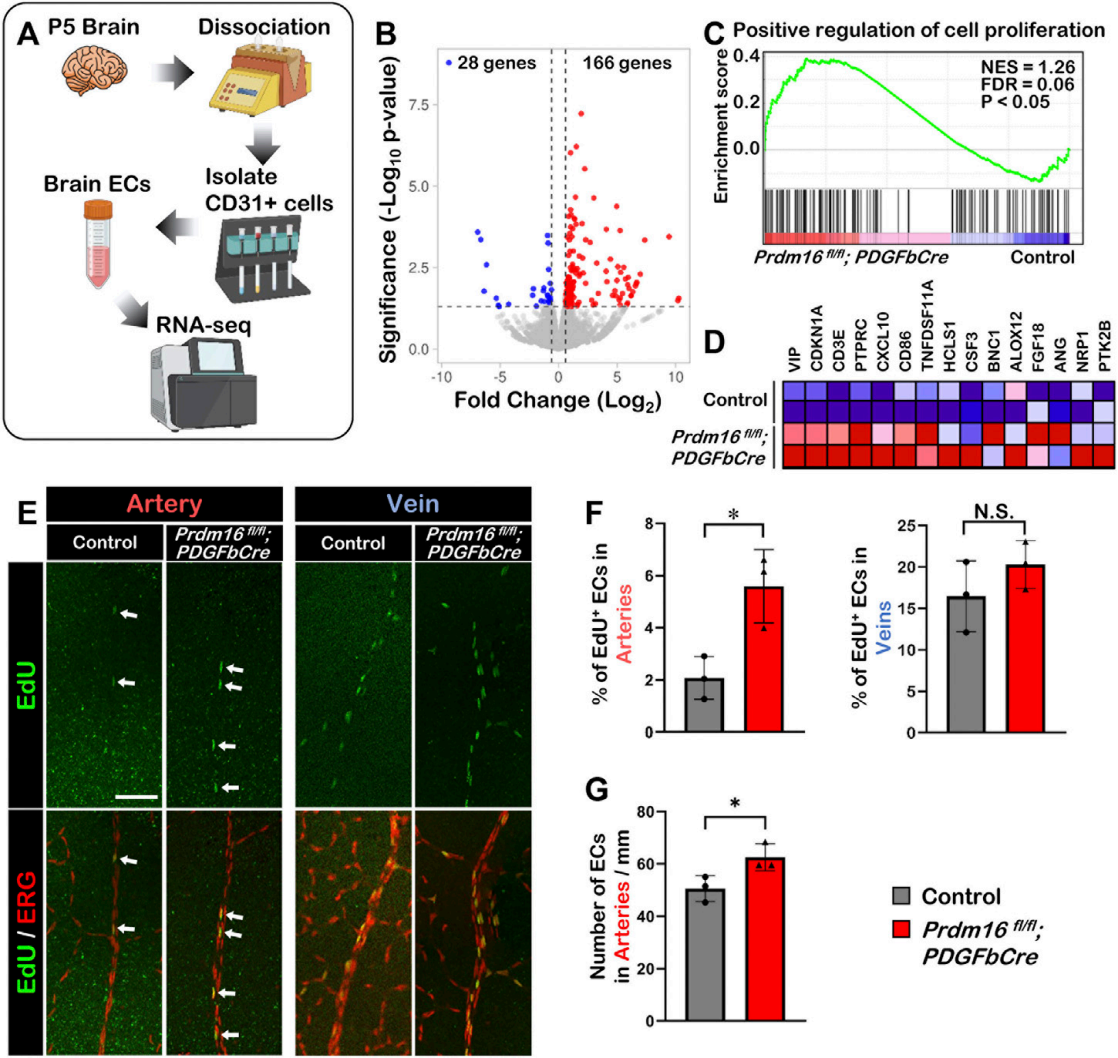
Loss of *Prdm16* leads to increased proliferation in arterial endothelial cells **(A)** Schematic for isolation of brain ECs from P5 mouse brains for RNA-seq. **(B)** Volcano plot of differentially expressed genes from P5 *Prdm16* ^*fl/fl*^ *; PDGFbCre* brain ECs. Blue dots represent downregulated genes, while red dots represent upregulated genes. Gray dots failed to meet minimum fold change of 1.5 or were not differentially expressed. **(C)** GSEA plot shows that cell proliferation genes were upregulated in *Prdm16* ^*fl/fl*^ *; PDGFbCre* ECs. **(D)** Heat map for top 15 genes in gene set. **(E)** Whole mount EdU staining of P8 retina. EdU (green) to label proliferating cells, ERG (red) used to mark EC nuclei. Scale bar, 100 µm. **(F)** Quantification of EdU^+^ ECs in arteries. Counted total number of EdU and ERG double positive cells in artery trunks until 50% extension, normalized to total number ERG single positive cells in the counted area. n = 3; Data are as mean ± SD, Student’s t-test: *p < 0.05. **(G)** Quantification of total number of ECs/mm in control and *Prdm16* ^*fl/fl*^ *; PDGFbCre* mouse retinal arteries. n = 3; Data are as mean ± SD, Student’s t-test: *p < 0.05.

To investigate the molecular function of PRDM16 during vascular development, we performed RNA-seq analysis using isolated ECs from P5 brains (Figure 6A). The volcano plot of the differential gene expression analysis showed that 166 genes were upregulated, and 28 genes were downregulated significantly in the *Prdm16* ^*fl/fl*^ *; PDGFbCre* ECs compared with those of littermate controls (Figure 6B). Since we observed ectopic venous marker expression in arterial ECs of *Prdm16* ^*fl/fl*^ *; PDGFbCre* retinas, we hypothesized that *Prdm16* KO arterial ECs may show some venous characteristics. We first focused on cell proliferation because EC proliferation in veins is generally higher than in arteries (McDonald et al., 2018; Sabbagh et al., 2018). GSEA analysis using a gene set for the “positive regulation of cell proliferation” showed that genes related to cell proliferation were significantly enriched in the *Prdm16* ^*fl/fl*^ *; PDGFbCre* brain ECs (Figures 6C, D). To examine the EC proliferation in the developing retinal vessels, 5-ethynyl-2′-deoxyuridin (EdU) was administered to P7 mice via IP injection 24 h before analysis. In contrast to only a few EdU-positive ECs detected in the arteries of control retinas, there were more EdU-positive arterial ECs in *Prdm16* ^*fl/fl*^ *; PDGFbCre* mice (Figures 6E, F). Consistently, we also observed an increased number of ECs in the arteries of the *Prdm16* ^*fl/fl*^ *; PDGFbCre* mice as compared with the control mice (Figure 6G). Note that there was no significant change of venous EC proliferation between control and *Prdm16* ^*fl/fl*^ *; PDGFbCre* retinas (Figures 6E, F), suggesting that PRDM16 might suppress the endothelial proliferation in the arteries during retinal vascular formation.

### Loss of *Prdm16* induces *Angpt2* expression in ECs

**FIGURE 7:**
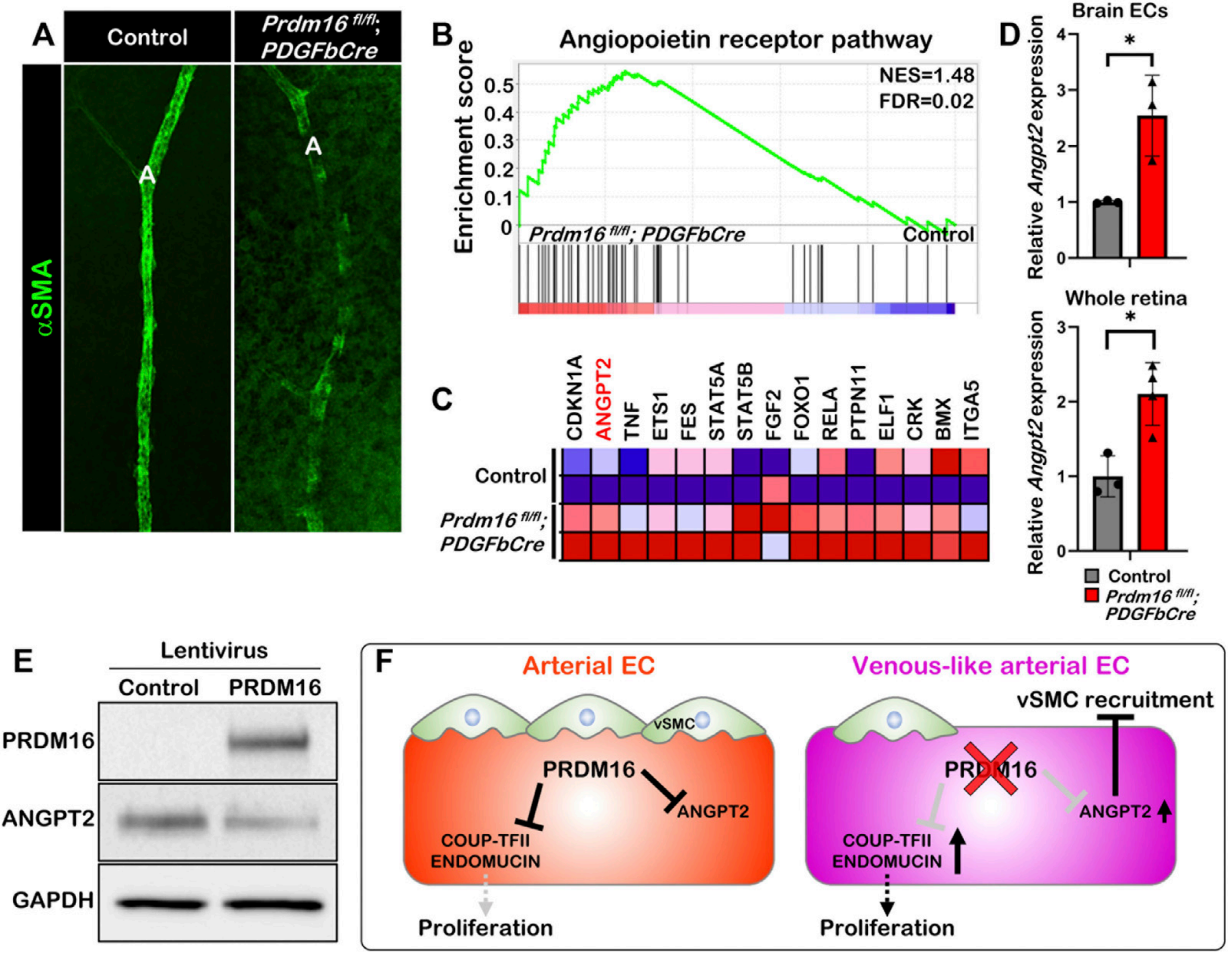
PRDM16 represses *Angpt2* expression in ECs **(A)** Whole mount αSMA immunostaining of control and *Prdm16* ^*fl/fl*^ *; PDGFbCre* retina at P9. αSMA, alpha smooth muscle actin (green). **(B)** GSEA plot shows that angiopoietin receptor pathway was upregulated in *Prdm16* ^*fl/fl*^ *; PDGFbCre* ECs. **(C)** Heat map of the top 15 dysregulated genes in Angiopoietin receptor pathway. **(D)** RT**-**qPCR analysis of *Angpt2* expression in P5 isolated brain ECs and P9 whole retina lysates. n = 3; Data are as mean ± SD, Student’s t-test: *p < 0.05. **(E)** Western blot of cell lysates from HUVECs infected with lentivirus expressing PRDM16 using antibodies against PRDM16, ANGPT2, and GAPDH. **(F)** Model for the role of PRDM16 in the maintenance of arterial EC characteristics. PRDM16 represses proliferation and the expression of venous markers in arterial ECs and inhibits *Angpt2* in arterial ECs, which allows vSMCs to associate to the arteries. Loss of PRDM16 leads to an increase in the proliferation of arterial ECs and the expression of venous markers and *Angpt2* leading to a reduction in vSMC recruitment to the arteries.

The ectopic venous marker expression in the arterial ECs of *Prdm16* mutant mice led us to further examine whether postnatal loss of *Prdm16* in ECs leads to a reduced capability to recruit vSMCs to arteries in the retina, as seen in the DAs of *Prdm16* ^*fl/fl*^ *; Tie2Cre* embryos. The immunostaining for αSMA showed less vSMC recruitment in the *Prdm16* ^*fl/fl*^ *; PDGFbCre* arteries (Figure 7A). To investigate a potential mechanism for the lack of vSMC recruitment seen in the *Prdm16* ^*fl/fl*^ *; Tie2Cre* embryos and *Prdm16* ^*fl/fl*^ *; PDGFbCre* retinas, we analyzed our RNA-seq data. Interestingly, GSEA analysis using a gene set for the angiopoietin receptor pathway showed that genes related to the angiopoietin pathway were significantly enriched in the *Prdm16* ^*fl/fl*^ *; PDGFbCre* brain ECs (Figures 7B, C). Furthermore, *Angpt2* was among the top 15 genes upregulated in the *Prdm16* ^*fl/fl*^ *; PDGFbCre* brain ECs as shown in the heatmap (Figure 7C). To confirm the upregulation of *Angpt2* expression, we performed RT-qPCR analysis using RNA from P5 isolated brain ECs or P9 whole retinas. The data showed that the expression of *Angpt2* was significantly increased in *Prdm16* ^*fl/fl*^ *; PDGFbCre* brain ECs and retinas (Figure 7D), suggesting that PRDM16 may act as a repressor for *Angpt2* expression in arterial ECs. Conversely, forced expression of PRDM16 in HUVECs was sufficient to drive arterial gene expression in the venous ECs (Supplementary Figure S3) and reduce the ANGPT2 level (Figure 7E). Together, these results suggest that PRDM16 promotes vSMC recruitment through suppression of *Angpt2* expression in arterial ECs (Figure 7F).

## Discussion

In the present study, we investigated the role of PRDM16 in ECs during vascular development. We found that loss of PRDM16 induced ectopic venous marker expression, less vSMC recruitment, and promoted cell proliferation in the arterial ECs. We also found that the *Prdm16* KO arterial ECs showed elevation of *Angpt2* expression that would prevent vSMC recruitment. These results strongly suggest that PRDM16 may play an important role in maintaining arterial characteristics.

At the beginning of vascular formation, immature ECs form the primitive vascular network, then arterial-venous specification occurs to mature the vessels (Swift and Weinstein, 2009). During the arterial-venous specification, immature ECs start to express arterial or venous markers. It is well known that the VEGF-Notch signaling pathway promotes arterial specific gene expression leading ECs towards an arterial fate (Corada et al., 2013). Conversely, the chicken ovalbumin upstream promoter transcription factor II (COUP-TFII/*Nr2f2*) has been reported to be expressed in venous ECs (Corada et al., 2013). ENDOMUCIN (encoded by *Emcn*) is an *O*-glycosylated single transmembrane sialomucin that is highly expressed in venous and capillary ECs, but not in the arterial ECs (Morgan et al., 1999; Kuhn et al., 2002). ENDOMUCIN was identified as a VEGF-induced angiogenic regulator (Park-Windhol et al., 2017). A previous *in vitro* study showed that loss of ENDOMUCIN function induces less EC migration, proliferation, and tube formation, while its over-expression enhances these phenotypes (Park-Windhol et al., 2017). We found that knockout of *Prdm16* in the ECs promotes ectopic ENDOMUCIN expression in the arterial ECs. Normally, arterial ECs in the retina show less proliferation while more proliferation is observed in venous ECs. Here we observed in the arterial ECs of *Prdm16* knockout retinas showed more proliferation compared to the control retinas, suggesting the possibility that the ectopic ENDOMUCIN expression may contribute to the increased cell proliferation in the *Prdm16* knockout arterial ECs.

We observed ectopic ENDOMUCIN and COUP-TFII expression in the *Prdm16* KO arterial ECs, but it remains unclear whether PRDM16 directly regulates *Emcn* or *Nr2f2* expression during vascular development. We predict that PRDM16 doesn’t have a strong ability to completely change the arterial-venous fate, but that it could be involved in the molecular mechanisms that maintain arterial characteristics. To elucidate the PRDM16 function during vascular formation, genome-wide chromatin immunoprecipitation sequencing (ChIP-seq) analysis is required.

Following the arterial-venous specification, arterial ECs start to recruit mural cells, such as pericytes and vSMCs, to stabilize vascular structure to sustain the arterial function (Gaengel et al., 2009). If the vSMC recruitment is compromised, blood vessels might become dilated and tend to be leaky (Hellstrom et al., 1999; Qin et al., 2013). It has been well characterized that the platelet-derived growth factor-B (PDGF-B) and the angiopoietins regulate paracrine interactions between ECs and vSMCs (Bergers and Song, 2005). Knockout of *Angpt1* (encoding ANGIOPOIETIN1) or its receptor *Tek* (encoding TIE2) induces embryonic lethality due to the failure of ECs to associate with the adjacent vSMCs. Transgenic mice overexpressing *Angpt2* showed a similar phenotype of *Angpt1* or *Tek* knockout embryos, indicating that ANGIOPOIETIN2 antagonizes TIE2 and competes for binding at the receptor with ANGIOPOIETIN1 (Maisonpierre et al., 1997). In this study, we found that *Angpt2* expression was upregulated in the arterial EC of *Prdm16* knockout retinas. Interestingly, consistent with *Angpt2* transgenic mice, *Prdm16* knockout embryos and retinas showed less vSMC recruitment to arterial ECs. To investigate whether PRDM16 directly regulates *Angpt2* expression, we examined available PRDM16 ChIP-seq data from heart ventricles (Wu et al., 2022) and found a peak upstream of the *Angpt2* transcription start site, strongly suggesting that PRDM16 promotes vSMC recruitment through the inhibition of *Angpt2* expression.

Although PRDM16 is only expressed in the arterial ECs, the *Prdm16* knockout retinas displayed less vascular formation entirely. It is also known that ANGIOPOIETIN1-TIE2 signaling pathway induces endothelial cell sprouting through the phosphatidylinositol 3′-kinase (PI3K)-AKT signaling pathway, and ANGIOPOIETIN2 disrupts vascular formation in the developing embryos (Maisonpierre et al., 1997; Morales-Ruiz et al., 2000). Since *Prdm16* knockout retinas showed upregulation of ANGPOIETIN2 expression, we hypothesize that paracrine ANGIPOIETIN2 signaling may also affect the neighboring PRDM16-negative ECs during vascular development. Deeper analysis for the activity of PI3K/AKT signaling pathway in the *Prdm16* knockout ECs is required to elucidate this question.

## Data Availability

The datasets presented in this study can be found in online repositories. The names of the repository/repositories and accession number(s) can be found below: https://www.ncbi.nlm.nih.gov/bioproject/946755.

## References

[B1] Aguilo F. Avagyan S. Labar A. Sevilla A. Lee D. F. Kumar P. (2011). Prdm16 is a physiologic regulator of hematopoietic stem cells. Blood 117 (19), 5057–5066. 10.1182/blood-2010-08-300145 21343612 PMC3109532

[B2] Becerril S. Gomez-Ambrosi J. Martin M. Moncada R. Sesma P. Burrell M. A. (2013). Role of Prdm16 in the activation of Brown fat programming. Relevance to the development of obesity. Histol. Histopathol. 28 (11), 1411–1425. 10.14670/HH-28.1411 23771475

[B3] Bergers G. Song S. (2005). The role of pericytes in blood-vessel formation and maintenance. Neuro Oncol. 7 (4), 452–464. 10.1215/s1152851705000232 16212810 PMC1871727

[B4] Biferali B. Bianconi V. Perez D. F. Kronawitter S. P. Marullo F. Maggio R. (2021). Prdm16-Mediated H3k9 methylation controls fibro-adipogenic progenitors identity during skeletal muscle repair. Sci. Adv. 7 (23). Epub 2021/06/04. 10.1126/sciadv.abd9371 PMC8172132 34078594

[B5] Birjiniuk A. Rosenfeld J. Tunuguntla H. Allen H. Penny D. Kim J. (2018). Deletions and loss of function mutations in Prdm16 are associated with pediatric cardiomyopathy. Circulation 138, 12162. 10.1161/circ.138.suppl_1.12162

[B6] Bjork B. C. Gomez A. C. Ahmed A. Aumann M. Jones J. Saadi I. (2018). Prdm16 and mecom mutants exhibit cleft secondary palate as a result of perturbations that affect different stages of palatogenesis. Faseb J. 32 (1), 776.7. 10.1096/fasebj.2018.32.1_supplement.776.7

[B7] Bjork B. C. Turbe-Doan A. Prysak M. Herron B. J. Beier D. R. (2010). Prdm16 is required for normal palatogenesis in mice. Hum. Mol. Genet. 19 (5), 774–789. 10.1093/hmg/ddp543 20007998 PMC2816611

[B8] Borensztein M. Viengchareun S. Montarras D. Journot L. Binart N. Lombes M. (2012). Double myod and Igf2 inactivation promotes Brown adipose tissue development by increasing Prdm16 expression. Faseb J. 26 (11), 4584–4591. 10.1096/fj.12-208496 22859371

[B9] Chen W. He S. Xiang D. (2021). Hypoxia-induced retinal pigment epithelium cell-derived bfgf promotes the migration and angiogenesis of huvecs through regulating tgf-ß1/smad2/3 pathway. Gene 790, 145695. Epub 20210505. 10.1016/j.gene.2021.145695 33964379

[B10] Chuikov S. Levi B. P. Smith M. L. Morrison S. J. (2010). Prdm16 promotes stem cell maintenance in multiple tissues, partly by regulating oxidative stress. Nat. Cell Biol. 12 (10), 999–1006. Epub 2010/09/14. 10.1038/ncb2101 20835244 PMC2948585

[B11] Cibi D. M. Bi-Lin K. W. Shekeran S. G. Sandireddy R. Tee N. Singh A. Prdm16 deficiency leads to age-dependent cardiac hypertrophy, adverse remodeling, mitochondrial dysfunction, and heart failure. Cell Rep. (2020) 33(3). 10.1016/j.celrep.2020.108288 33086060

[B12] Claxton S. Kostourou V. Jadeja S. Chambon P. Hodivala-Dilke K. Fruttiger M. (2008). Efficient, inducible cre-recombinase activation in vascular endothelium. Genesis 46 (2), 74–80. Epub 2008/02/08. 10.1002/dvg.20367 18257043

[B13] Cohen P. Levy J. D. Zhang Y. Y. Frontini A. Kolodin D. P. Svensson K. J. (2014). Ablation of Prdm16 and beige adipose causes metabolic dysfunction and a subcutaneous to visceral fat switch. Cell 156 (1-2), 304–316. 10.1016/j.cell.2013.12.021 24439384 PMC3922400

[B14] Corada M. Orsenigo F. Morini M. F. Pitulescu M. E. Bhat G. Nyqvist D. (2013). Sox17 is indispensable for acquisition and maintenance of arterial identity. Nat. Commun. 4, 2609. Epub 2013/10/25. 10.1038/ncomms3609 24153254 PMC3826640

[B15] Corrigan D. J. Luchsinger L. L. de Almeida M. J. Williams L. J. Strikoudis A. Snoeck H. W. (2018). Prdm16 isoforms differentially regulate normal and leukemic hematopoiesis and inflammatory gene signature. J. Clin. Investigation 128 (8), 3250–3264. 10.1172/Jci99862 PMC6063481 29878897

[B16] Craps S. Van Wauwe J. De Moudt S. De Munck D. Leloup A. J. A. Boeckx B. (2021). Prdm16 supports arterial flow recovery by maintaining endothelial function. Circulation Res. 129 (1), 63–77. 10.1161/Circresaha.120.318501 33902304 PMC8221541

[B17] Crist A. M. Young C. Meadows S. M. (2017). Characterization of arteriovenous identity in the developing neonate mouse retina. Gene Expr. Patterns 23-24, 22–31. Epub 2017/02/09. 10.1016/j.gep.2017.01.002 28167138

[B18] Cunha S. I. Magnusson P. U. Dejana E. Lampugnani M. G. (2017). Deregulated tgf-Β/bmp signaling in vascular malformations. Circulation Res. 121, 981–999. 10.1161/CIRCRESAHA.117.309930 28963191

[B19] Dumont D. J. Gradwohl G. Fong G. H. Puri M. C. Gertsenstein M. Auerbach A. (1994). Dominant-negative and targeted null mutations in the endothelial receptor tyrosine kinase, Tek, reveal a critical role in vasculogenesis of the embryo. Genes Dev. 8 (16), 1897–1909. 10.1101/gad.8.16.1897 7958865

[B20] Fischer A. Schumacher N. Maier M. Sendtner M. Gessler M. (2004). The Notch target genes Hey1 and Hey2 are required for embryonic vascular development. Genes Dev. 18 (8), 901–911. 10.1101/gad.291004 15107403 PMC395849

[B21] Fog C. K. Galli G. G. Lund A. H. (2012). Prdm proteins: Important players in differentiation and disease. Bioessays 34 (1), 50–60. Epub 2011/10/27. 10.1002/bies.201100107 22028065

[B22] Gaengel K. Genové G. Armulik A. Betsholtz C. (2009). Endothelial-mural cell signaling in vascular development and angiogenesis. Arteriosclerosis, Thrombosis, Vasc. Biol. 29, 630–638. 10.1161/ATVBAHA.107.161521 19164813

[B23] Gale N. W. Thurston G. Hackett S. F. Renard R. Wang Q. McClain J. (2002). Angiopoietin-2 is required for postnatal angiogenesis and lymphatic patterning, and only the latter role is rescued by angiopoietin-1. Dev. Cell 3 (3), 411–423. 10.1016/s1534-5807(02)00217-4 12361603

[B24] Gan L. Liu Z. Feng F. Wu T. Luo D. Hu C. (2018). Foxc2 coordinates inflammation and browning of white adipose by leptin-stat3-prdm16 signal in mice. Int. J. Obes. 42 (2), 252–259. 10.1038/ijo.2017.208 28925407

[B25] Gerhardt H. Golding M. Fruttiger M. Ruhrberg C. Lundkvist A. Abramsson A. (2003). Vegf guides angiogenic sprouting utilizing endothelial tip cell filopodia. J. Cell Biol. 161 (6), 1163–1177. Epub 2003/06/16. 10.1083/jcb.200302047 12810700 PMC2172999

[B26] Goldie L. C. Nix M. K. Hirschi K. K. (2008). Embryonic vasculogenesis and hematopoietic specification. Organogenesis 4 (4), 257–263. 10.4161/org.4.4.7416 19337406 PMC2634331

[B27] Gridley T. (2007). Notch signaling in vascular development and Physiology. Development 134 (15), 2709–2718. 10.1242/dev.004184 17611219

[B28] Harms M. J. Ishibashi J. Wang W. S. Lim H. W. Goyama S. Sato T. (2014). Prdm16 is required for the maintenance of Brown adipocyte identity and function in adult mice. Cell Metab. 19 (4), 593–604. 10.1016/j.cmet.2014.03.007 24703692 PMC4012340

[B29] Hellstrom M. Kal N. M. Lindahl P. Abramsson A. Betsholtz C. (1999). Role of pdgf-B and pdgfr-beta in recruitment of vascular smooth muscle cells and pericytes during embryonic blood vessel formation in the mouse. Development 126 (14), 3047–3055. 10.1242/dev.126.14.3047 10375497

[B30] Jeong H. W. Hernández-Rodríguez B. Kim J. Kim K. P. Enriquez-Gasca R. Yoon J. (2017). Transcriptional regulation of endothelial cell behavior during sprouting angiogenesis. Nat. Commun. 8 (1), 726. Epub 20170928. 10.1038/s41467-017-00738-7 28959057 PMC5620061

[B31] Kisanuki Y. Y. Hammer R. E. Miyazaki J. Williams S. C. Richardson J. A. Yanagisawa M. (2001). Tie2-Cre transgenic mice: A new model for endothelial cell-lineage analysis *in vivo* . Dev. Biol. 230 (2), 230–242. 10.1006/dbio.2000.0106 11161575

[B32] Krebs L. T. Xue Y. Norton C. R. Shutter J. R. Maguire M. Sundberg J. P. (2000). Notch signaling is essential for vascular morphogenesis in mice. Genes Dev. 14 (11), 1343–1352. 10.1101/gad.14.11.1343 10837027 PMC316662

[B33] Kuhn A. Brachtendorf G. Kurth F. Sonntag M. Samulowitz U. Metze D. (2002). Expression of endomucin, a novel endothelial sialomucin, in normal and diseased human skin. J. Invest. Dermatol 119 (6), 1388–1393. 10.1046/j.1523-1747.2002.19647.x 12485444

[B34] Kume T. (2010). Specification of arterial, venous, and lymphatic endothelial cells during embryonic development. Histol. Histopathol. 25 (5), 637–646. 10.14670/HH-25.637 20238301 PMC2899674

[B36] Levéen P. Pekny M. Gebre-Medhin S. Swolin B. Larsson E. Betsholtz C. (1994). Mice deficient for pdgf B show renal, cardiovascular, and hematological abnormalities. Genes Dev. 8 (16), 1875–1887. 10.1101/gad.8.16.1875 7958863

[B37] Li X. Wang J. Q. Jiang Z. Guo F. Soloway P. D. Zhao R. Q. (2015). Role of Prdm16 and its Pr domain in the epigenetic regulation of myogenic and adipogenic genes during transdifferentiation of C2c12 cells. Gene 570 (2), 191–198. 10.1016/j.gene.2015.06.017 26071185

[B38] Lindahl P. Johansson B. R. Leveen P. Betsholtz C. (1997). Pericyte loss and microaneurysm formation in pdgf-B-deficient mice. Science 277 (5323), 242–245. Epub 1997/07/11. 10.1126/science.277.5323.242 9211853

[B39] Ma J. Tang W. Gu R. Hu F. Zhang L. Wu J. (2020). Shp-2-Induced activation of C-myc is involved in pdgf-B-regulated cell proliferation and angiogenesis in rmecs. Front. Physiol. 11, 555006. Epub 20201123. 10.3389/fphys.2020.555006 33329018 PMC7719712

[B40] Maisonpierre P. C. Suri C. Jones P. F. Bartunkova S. Wiegand S. J. Radziejewski C. (1997). Angiopoietin-2, a natural antagonist for Tie2 that disrupts *in vivo* angiogenesis. Science 277 (5322), 55–60. 10.1126/science.277.5322.55 9204896

[B41] Matrone G. Xia B. Chen K. Denvir M. A. Baker A. H. Cooke J. P. (2021). Fli1(+) cells transcriptional analysis reveals an lmo2-prdm16 Axis in angiogenesis. Proc. Natl. Acad. Sci. U. S. A. 118 (31). Epub 2021/08/01. 10.1073/pnas.2008559118 PMC8346798 34330825

[B42] McDonald A. I. Shirali A. S. Aragón R. Ma F. Hernandez G. Vaughn D. A. (2018). Endothelial regeneration of large vessels is a biphasic process driven by local cells with distinct proliferative capacities. Cell Stem Cell 23 (2), 210–225.e6. 10.1016/j.stem.2018.07.011 30075129 PMC6178982

[B43] Morales-Ruiz M. Fulton D. Sowa G. Languino L. R. Fujio Y. Walsh K. (2000). Vascular endothelial growth factor-stimulated actin reorganization and migration of endothelial cells is regulated via the serine/threonine kinase akt. Circ. Res. 86 (8), 892–896. 10.1161/01.res.86.8.892 10785512

[B44] Morgan S. M. Samulowitz U. Darley L. Simmons D. L. Vestweber D. (1999). Biochemical characterization and molecular cloning of a novel endothelial-specific sialomucin. Blood 93 (1), 165–175. 9864158

[B45] Nam J. M. Lim J. E. Ha T. W. Oh B. Kang J. O. (2020). Cardiac-specific inactivation of Prdm16 effects cardiac conduction abnormalities and cardiomyopathy-associated phenotypes. Am. J. Physiol. Heart Circ. Physiol. 318 (4), H764–H77. Epub 2020/02/23. 10.1152/ajpheart.00647.2019 32083975

[B46] Park-Windhol C. Ng Y. S. Yang J. Primo V. Saint-Geniez M. D’Amore P. A. (2017). Endomucin inhibits vegf-induced endothelial cell migration, growth, and morphogenesis by modulating Vegfr2 signaling. 10.1038/s41598-017-16852-x PMC5719432 29215001

[B47] Pinheiro I. Margueron R. Shukeir N. Eisold M. Fritzsch C. Richter F. M. (2012). Prdm3 and Prdm16 are H3k9me1 methyltransferases required for mammalian heterochromatin integrity. Cell 150 (5), 948–960. 10.1016/j.cell.2012.06.048 22939622

[B48] Qin D. Trenkwalder T. Lee S. Chillo O. Deindl E. Kupatt C. (2013). Early vessel destabilization mediated by angiopoietin-2 and subsequent vessel maturation via angiopoietin-1 induce functional neovasculature after ischemia. PLoS One 8 (4), e61831. Epub 20130416. 10.1371/journal.pone.0061831 23613948 PMC3628915

[B49] Rapp S. J. Dershem V. Zhang X. Schutte S. C. Chariker M. E. (2020). Varying negative pressure wound therapy acute effects on human split-thickness autografts. J. Burn Care Res. 41 (1), 104–112. 10.1093/jbcr/irz122 31420676

[B50] Sabbagh M. F. Heng J. S. Luo C. Castanon R. G. Nery J. R. Rattner A. (2018). Transcriptional and epigenomic landscapes of cns and non-cns vascular endothelial cells. Elife 7, e36187. Epub 20180906. 10.7554/eLife.36187 30188322 PMC6126923

[B51] Sakabe M. Fan J. Odaka Y. Liu N. Hassan A. Duan X. (2017). Yap/Taz-Cdc42 signaling regulates vascular tip cell migration. Proc. Natl. Acad. Sci. U. S. A. 114 (41), 10918–10923. Epub 2017/10/05. 10.1073/pnas.1704030114 28973878 PMC5642684

[B52] Sato T. N. Tozawa Y. Deutsch U. Wolburg-Buchholz K. Fujiwara Y. Gendron-Maguire M. (1995). Distinct roles of the receptor tyrosine kinases tie-1 and tie-2 in blood vessel formation. Nature 376 (6535), 70–74. 10.1038/376070a0 7596437

[B53] Schindelin J. Arganda-Carreras I. Frise E. Kaynig V. Longair M. Pietzsch T. (2012). Fiji: An open-source platform for biological-image analysis. Nat. Methods 9 (7), 676–682. 10.1038/nmeth.2019 22743772 PMC3855844

[B54] Schneider C. A. Rasband W. S. Eliceiri K. W. (2012). Nih image to imagej: 25 Years of image analysis. Nat. Methods 9 (7), 671–675. Epub 2012/08/30. 10.1038/nmeth.2089 22930834 PMC5554542

[B55] Seale P. Kajimura S. Yang W. Chin S. Rohas L. M. Uldry M. (2007). Transcriptional control of Brown fat determination by Prdm16. Cell Metab. 6 (1), 38–54. Epub 2007/07/10. 10.1016/j.cmet.2007.06.001 17618855 PMC2564846

[B56] Shih S. C. Ju M. Liu N. Mo J. R. Ney J. J. Smith L. E. (2003). Transforming growth factor Beta1 induction of vascular endothelial growth factor receptor 1: Mechanism of pericyte-induced vascular survival *in vivo* . Proc. Natl. Acad. Sci. U. S. A. 100 (26), 15859–15864. Epub 20031203. 10.1073/pnas.2136855100 14657382 PMC307658

[B57] Shull L. C. Sen R. Menzel J. Goyama S. Kurokawa M. Artinger K. B. (2020). The conserved and divergent roles of Prdm3 and Prdm16 in zebrafish and mouse craniofacial development. Dev. Biol. 461 (2), 132–144. Epub 2020/02/12. 10.1016/j.ydbio.2020.02.006 32044379 PMC7198358

[B58] Stratman A. N. Burns M. C. Farrelly O. M. Davis A. E. Li W. Pham V. N. (2020). Chemokine mediated signalling within arteries promotes vascular smooth muscle cell recruitment. Commun. Biol. 3 (1), 734. Epub 20201204. 10.1038/s42003-020-01462-7 33277595 PMC7719186

[B59] Su L. B. Lei X. P. Ma H. Y. Feng C. Jiang J. Jiao J. W. (2020). Prdm16 orchestrates angiogenesis via neural differentiation in the developing brain. Cell Death Differ. 27 (8), 2313–2329. 10.1038/s41418-020-0504-5 32015502 PMC7370227

[B60] Suri C. Jones P. F. Patan S. Bartunkova S. Maisonpierre P. C. Davis S. (1996). Requisite role of angiopoietin-1, a ligand for the Tie2 receptor, during embryonic angiogenesis. Cell 87 (7), 1171–1180. 10.1016/s0092-8674(00)81813-9 8980224

[B61] Swift M. R. Weinstein B. M. (2009). Arterial-venous specification during development. Circulation Res. 104 (5), 576–588. 10.1161/Circresaha.108.188805 19286613

[B62] Walsh K. B. Zhang X. Zhu X. Wohleb E. Woo D. Lu L. (2019). Intracerebral hemorrhage induces inflammatory gene expression in peripheral blood: Global transcriptional profiling in intracerebral hemorrhage patients. DNA Cell Biol. 38 (7), 660–669. Epub 20190522. 10.1089/dna.2018.4550 31120332 PMC6909779

[B63] Wu T. Liang Z. Zhang Z. Liu C. Zhang L. Gu Y. (2022). Prdm16 is a compact myocardium-enriched transcription factor required to maintain compact myocardial cardiomyocyte identity in left ventricle. Circulation 145 (8), 586–602. Epub 20211217. 10.1161/CIRCULATIONAHA.121.056666 34915728 PMC8860879

[B64] You L. R. Lin F. J. Lee C. T. DeMayo F. J. Tsai M. J. Tsai S. Y. (2005). Suppression of Notch signalling by the coup-tfii transcription factor regulates vein identity. Nature 435 (7038), 98–104. Epub 2005/05/06. 10.1038/nature03511 15875024

[B65] Zudaire E. Gambardella L. Kurcz C. Vermeren S. (2011). A computational tool for quantitative analysis of vascular networks. PLoS One 6 (11), e27385. Epub 20111116. 10.1371/journal.pone.0027385 22110636 PMC3217985

