# Supplementary material for: PRDM16 regulates arterial development and vascular integrity
Source: Front Physiol. 2023 Jun 1;14:1165379. doi: 10.3389/fphys.2023.1165379 (PMC10267475; doi:10.3389/fphys.2023.1165379)
Supplement: Supplementary file 1 [file Image1.pdf]

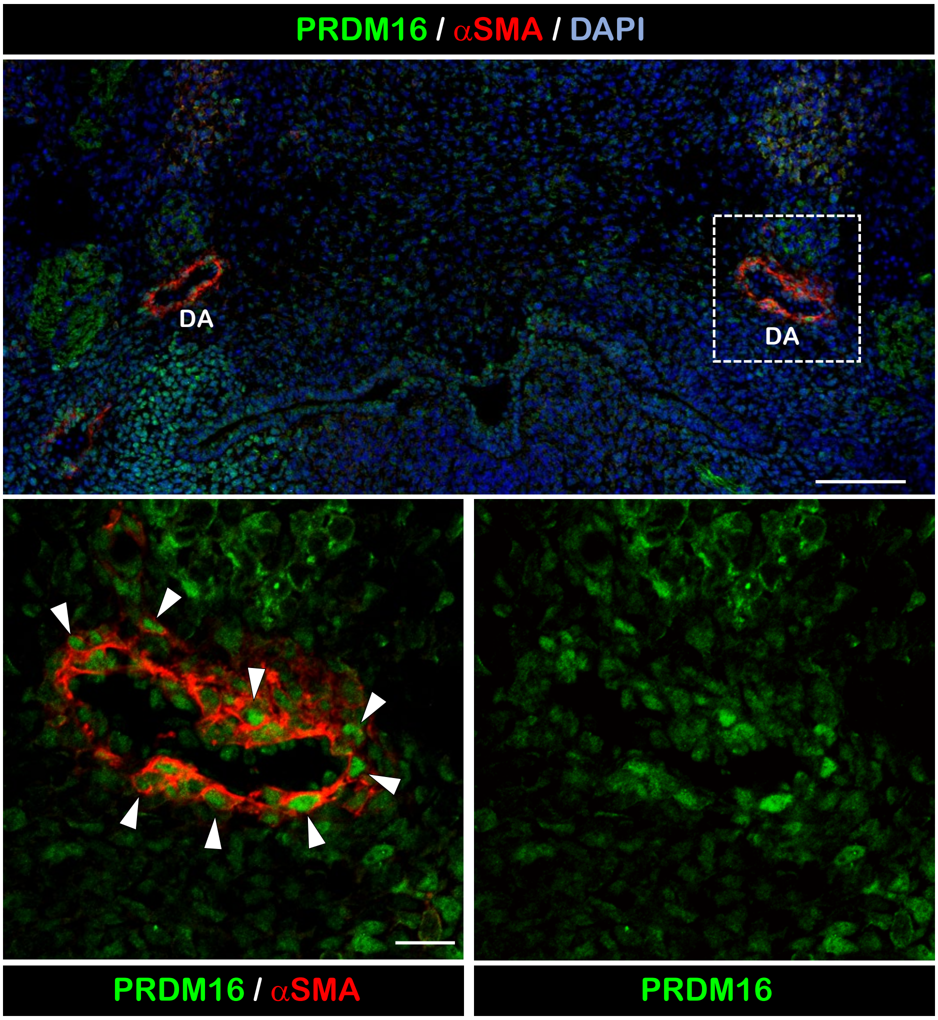

Supplemental Figure S1: PRDM16 is present in the vSMCs of DA
PRDM16 immunostaining of transverse section of WT E11.5 mouse embryo. PRDM16 (green) vascular smooth muscle cells (αSMA, red). DA, dorsal aorta. Scale bars, 100µm (upper panel); 20µm (lower panel).

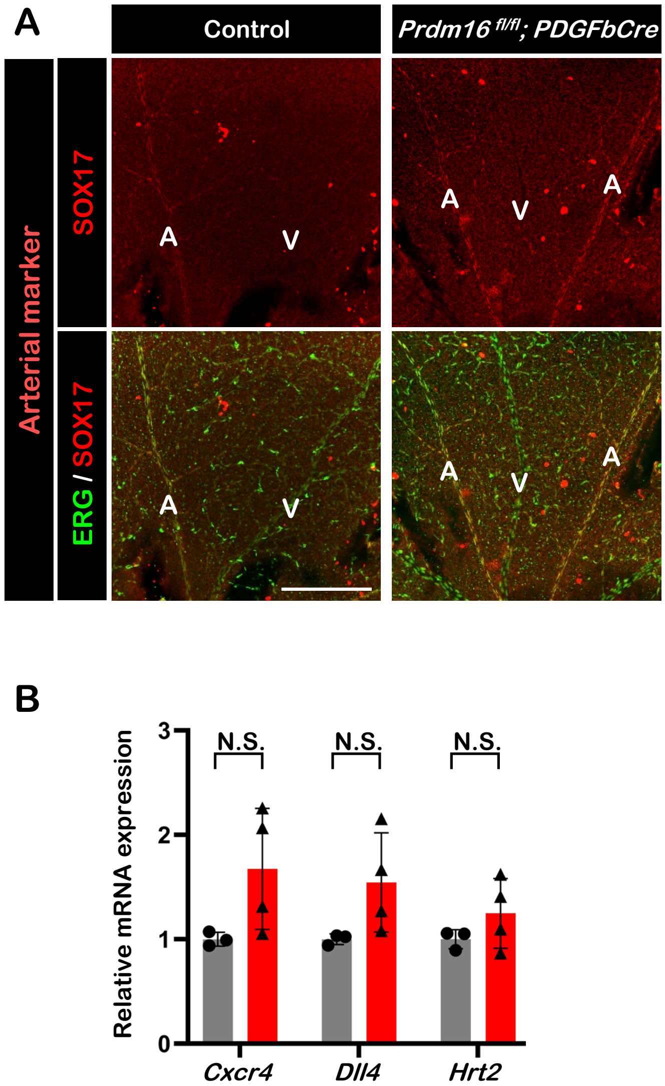

Supplemental Figure S2: Loss of *Prdm16* does not change expression of arterial markers (A) SOX17 immunostaining of whole mount P10 retinas of the control and *Prdm16*$^{fl/fl}$*; PDGFbCre* mice (SOX17, red). (ERG, green) n≥3. Scale bar, 100μm. (B) RT-qPCR analysis for arterial marker genes *Cxcr4*, *Dll4*, and *Hrt2* from the whole retina. n≥3. Data are shown as mean ± SD, Student t test: ns

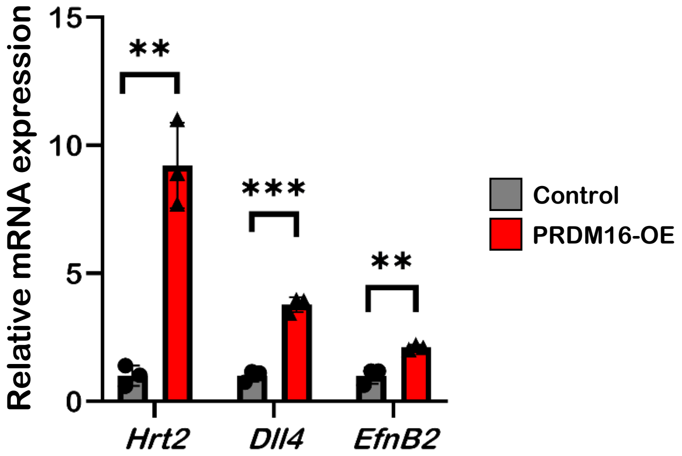

Supplemental Figure S3: Overexpression of PRDM16 in HUVECs promotes arterial marker expression

RT-qPCR analysis of arterial markers *Hrt2*, *Dll4*, *EphB2* in HUVECs infected with control or *Prdm16* lentivirus (PRDM16-OE). n=3. Data are shown as mean ± SD, Student t test: **$p<0.01$, ***$p<0.001$.

**Table 1** Antibody Information			
**Antibody/Stain**	**Dilution**	**Manufacturer**	**Part #**
Sheep anti-PRDM16	1:200	R&D Systems	AF6295-SP
Rat anti-PECAM1	1:100	BD Biosciences	553369
DAPI	1:1000	ThermoFisher	D1306
Goat anti-SOX17	1:100	R&D Systems	AF1924
Cy3 conjugated anti-αSMA	1:100	Sigma	C6198
Rabbit anti-ERG1/2/3	1:100	abcam	ab92513
Donkey anti-rat 594	1:200	ThermoFisher	A21209
Donkey anti-sheep 488	1:200	Invitrogen	A11015
Donkey anti-Goat 488	1:200	Invitrogen	A11055
Donkey anti-Rat 594	1:200	ThermoFisher	A21209
Donkey anti-rat 488	1:200	ThermoFisher	A21208
Donkey anti-sheep Cy3	1:200	Jackson ImmunoResearch Laboratories	713-165-003
Donkey anti-rabbit 488	1:200	ThermoFisher	A21206
Anti-sheep HRP	1:1000	Sigma	A3415
Anti-mouse HRP	1:1000	GE Healthcare	NA931-1ML
Mouse anti-GAPDH	1:3000	Millipore	MAB374
Goat anti-ANGPT2	1:500	R&D Systems	AF623
Anti-goat HRP	1:5000	Millipore	401515
Rat anti-ENDOMUCIN	1:100	SantaCruz	sc-65495
Mouse anti-COUP-TFII	1:100	R&D Systems	PP-H7147-00

**Table 2** Primer Information		
**Gene Name**	**Forward Primer Sequence (5' → 3')**	**Reverse Primer Sequence (5' → 3')**
Angpt2	TTAGCACAAAGGATTCGGACAAT	TTTTGTGGGTAGTACTGTCCATTCA
Pecam1	GGTGCATGGCGTATCCAAG	TGGAGGTCTTATCTATCCTTCGC
Hrt2	AAAGGCGTCGGGATCGGATA	AGAGCGTGTGCGTCAAAGTAG
Dll4	GCCCTTCAATTTCACCTGGC	CAATAACCAGTTCTGACCCACAG
EfnB2	TATGCAGAACTGCGATTTCCAA	TGGGTATAGTACCAGTCCTTGTC
Cxcr4	ACTACACCGAGGAAATGGGCT	CCCACAATGCCAGTTAAGAAGA
18s	GTCTGTGATGCCCTTAGATG	AGCTTATGACCCGCACTTAC
